# Characterization of VPS34-IN1, a selective inhibitor of Vps34, reveals that the phosphatidylinositol 3-phosphate-binding SGK3 protein kinase is a downstream target of class III phosphoinositide 3-kinase

**DOI:** 10.1042/BJ20140889

**Published:** 2014-10-10

**Authors:** Ruzica Bago, Nazma Malik, Michael J. Munson, Alan R. Prescott, Paul Davies, Eeva Sommer, Natalia Shpiro, Richard Ward, Darren Cross, Ian G. Ganley, Dario R. Alessi

**Affiliations:** *MRC Protein Phosphorylation and Ubiquitylation Unit, College of Life Sciences, University of Dundee, Dundee DD1 5EH, U.K.; †Division of Cell Signalling and Immunology, College of Life Sciences, University of Dundee, Dundee DD1 5EH, U.K.; ‡Oncology iMED, AstraZeneca, Alderley Park, Cheshire SK10 4TG, U.K.

**Keywords:** mammalian target of rapamycin (mTOR), N-Myc downstream-regulated gene-1 (NDRG1), phosphoinositide 3-kinase (PI3K), protein kinase inhibitor, signal transduction inhibitor, 4E-BP1, eukaryotic initiation factor 4E-binding protein 1, DMEM, Dulbecco's modified Eagle's medium, EEA1, early endosome antigen 1, HRP, horseradish peroxidase, IGF, insulin-like growth factor, INPP4B, inositol polyphosphate 4-phosphatase type II, IP1, inositol phosphate, ITC, isothermal titration calorimetry, mTOR, mammalian target of rapamycin, mTORC, mammalian target of rapamycin complex, NDRG1, N-Myc downstream-regulated gene-1, PDK1, phosphoinositide-dependent kinase 1, PH, pleckstrin homology, PI3K, phosphoinositide 3-kinase, PRAS40, proline-rich Akt substrate 40 kDa, PtdIns, phosphatidylinositol, PX, Phox homology, SGK3, serum- and glucocorticoid-regulated kinase-3, SHIP1/2, Src homology 2-domain-containing inositol phosphatase 1/2, TSC2, tuberous sclerosis complex 2, Vps34, vacuolar protein sorting 34, VSV-G, vesicular stomatitis virus glycoprotein

## Abstract

The Vps34 (vacuolar protein sorting 34) class III PI3K (phosphoinositide 3-kinase) phosphorylates PtdIns (phosphatidylinositol) at endosomal membranes to generate PtdIns(3)*P* that regulates membrane trafficking processes via its ability to recruit a subset of proteins possessing PtdIns(3)*P*-binding PX (phox homology) and FYVE domains. In the present study, we describe a highly selective and potent inhibitor of Vps34, termed VPS34-IN1, that inhibits Vps34 with 25 nM IC_50_ *in vitro*, but does not significantly inhibit the activity of 340 protein kinases or 25 lipid kinases tested that include all isoforms of class I as well as class II PI3Ks. Administration of VPS34-IN1 to cells induces a rapid dose-dependent dispersal of a specific PtdIns(3)*P*-binding probe from endosome membranes, within 1 min, without affecting the ability of class I PI3K to regulate Akt. Moreover, we explored whether SGK3 (serum- and glucocorticoid-regulated kinase-3), the only protein kinase known to interact specifically with PtdIns(3)*P* via its N-terminal PX domain, might be controlled by Vps34. Mutations disrupting PtdIns(3)*P* binding ablated SGK3 kinase activity by suppressing phosphorylation of the T-loop [PDK1 (phosphoinositide-dependent kinase 1) site] and hydrophobic motif (mammalian target of rapamycin site) residues. VPS34-IN1 induced a rapid ~50–60% loss of SGK3 phosphorylation within 1 min. VPS34-IN1 did not inhibit activity of the SGK2 isoform that does not possess a PtdIns(3)*P*-binding PX domain. Furthermore, class I PI3K inhibitors (GDC-0941 and BKM120) that do not inhibit Vps34 suppressed SGK3 activity by ~40%. Combining VPS34-IN1 and GDC-0941 reduced SGK3 activity ~80–90%. These data suggest SGK3 phosphorylation and hence activity is controlled by two pools of PtdIns(3)*P*. The first is produced through phosphorylation of PtdIns by Vps34 at the endosome. The second is due to the conversion of class I PI3K product, PtdIns(3,4,5)*P*_3_ into PtdIns(3)*P*, via the sequential actions of the PtdIns 5-phosphatases [SHIP1/2 (Src homology 2-domain-containing inositol phosphatase 1/2)] and PtdIns 4-phosphatase [INPP4B (inositol polyphosphate 4-phosphatase type II)]. VPS34-IN1 will be a useful probe to delineate physiological roles of the Vps34. Monitoring SGK3 phosphorylation and activity could be employed as a biomarker of Vps34 activity, in an analogous manner by which Akt is used to probe cellular class I PI3K activity. Combining class I (GDC-0941) and class III (VPS34-IN1) PI3K inhibitors could be used as a strategy to better analyse the roles and regulation of the elusive class II PI3K.

## Abstract 2

We characterize VPS34-IN, a potent and selective inhibitor of class III Vps34 PI3K. Using VPS34-IN1, we demonstrate that PtdIns(3)*P*, produced by Vps34 controls phosphorylation and activity of the SGK3 protein kinase.

## INTRODUCTION

PI3Ks (phosphoinositide 3-kinases) phosphorylate the 3′-position hydroxy group of the D-*myo*-inositol head group of PtdIns (phosphatidylinositol) to generate 3-phosphoinositides that are critical in switching on downstream signalling pathways [1]. These orchestrate a wide range of biological responses including controlling cell growth, proliferation and intracellular trafficking [2,3]. Work in this area has taken on added urgency, as it is now clear that understanding disruptions in diverse PI3K signalling pathways lie at the centre of understanding major diseases such as cancer, inflammation heart failure and diabetes [4–6].

There are three different classes of PI3Ks termed class I, class II and class III [7]. These enzymes act on most cellular membranes. The most studied enzymes are the four members of class I PI3Ks (p110α, p110β p110γ and p110δ) that phosphorylate PtdIns(4,5)*P*_2_ at the plasma membrane to generate the signalling second messenger PtdIns(3,4,5)*P*_3_ in response to agonists that trigger activation of growth factors, Ras or G-protein-coupled receptors [8]. PtdIns(3,4,5)*P*_3_, as well as its immediate breakdown product PtdIns(3,4)*P*_2_, trigger downstream signalling responses by interacting specifically with a subgroup of signalling proteins that possess a PH (pleckstrin homology) domain that bind with high affinity to these 3-phosphoinositides [9]. One of the best characterized signalling pathways activated by class I PI3K proteins are the Akt protein kinases that control critical processes such as metabolism and growth by phosphorylating multiple targets including FOXO (forkhead box O) transcription factors, TSC2 (tuberous sclerosis complex 2) and GSK3 (glycogen synthase kinase 3) [10]. Following activation of class I PI3Ks, Akt and one of its upstream protein kinases termed PDK1 (phosphoinositide-dependent kinase 1) are recruited to the plasma membrane via their PtdIns(3,4,5)*P*_3_/PtdIns(3,4)*P*_2_-binding PH domains. This results in PDK1 phosphorylating the T-loop Thr^308^ residue thereby partially activating Akt [11–14]. Akt is also phosphorylated by mTORC2 (mammalian target of rapamycin complex-2) at Ser^473^ located within the hydrophobic motif at the C-terminal non-catalytic region [15]. Phosphorylation of Ser^473^ also serves to promote the phosphorylation of Akt at Thr^308^ by PDK1 [16]. PtdIns(3,4,5)*P*_3_ can then be converted into PtdIns(3)*P* via the sequential actions of the PtdIns 5-phosphatases [SHIP1/2 (Src homology 2-domain-containing inositol phosphatase 1/2)] [17] and PtdIns 4-phosphatase [INPP4B (inositol polyphosphate 4-phosphatase type II)] [18]. PtdIns(3)*P* is rapidly dephosphorylated in cells to PtdIns by a family of myotubularin PtdIns 3-phosphatases [19].

Class II and class III PI3Ks phosphorylate PtdIns to generate PtdIns(3)*P*. The class II PI3K subfamily has three members in vertebrates (PI3KC2α, PI3KC2β and PI3KC2γ), but the roles that these perform and how they are regulated are poorly understood [20,21]. The single class III PI3K isoform termed Vps34 (vacuolar protein sorting 34) play an important role in controlling vesicular protein sorting, a phenomenon that was first discovered in yeast [22]. In all eukaryotes, Vps34 forms a core complex with two other protein subunits termed Vps15 (a serine/threonine protein kinase) [23] and beclin-1 (also known as Vps30 or ATG6) [24]. This core complex then interacts with a growing list of proteins to form distinct complexes controlling membrane endosomal trafficking processes and endosome–lysosome maturation as well as autophagy [25,26].

The most thoroughly characterized PtdIns(3)*P*-binding domains are a subset of FYVE and PX (Phox homology) domains [27–30]. There is a pool of PtdIns(3)*P* that is highly enriched on early endosomes and in the internal vesicles of multivesicular endosomes [31]. PtdIns(3)*P*-binding PX and FYVE domains display relatively low PtdIns(3)*P*-binding affinity which, in combination with high myotubularin PtdIns 3-phosphatase activity, permits rapid and highly dynamic localization and responses [25,29]. Consistent with a key role of Vps34 and PtdIns(3)*P* in regulating vesicular trafficking, several proteins containing PX and FYVE domains specific for PtdIns(3)*P* have been identified that play critical roles in protein sorting pathways [32].

In the present study, we characterize a Vps34 inhibitor termed VPS34-IN1. We show that this compound inhibits recombinant Vps34 with nanomolar potency, but does not significantly inhibit other protein kinases or lipid kinases tested, including class I or class II PI3Ks. We demonstrate that VPS34-IN1 rapidly reduces endosomal PtdIns(3)*P* levels within 1 min of drug treatment. Furthermore, we utilize VPS34-IN1 to demonstrate that Vps34 plays a role in regulating the phosphorylation and activity of the SGK3 (serum- and glucocorticoid-regulated protein kinase-3), which is the only protein kinase known to possess a selective PtdIns(3)*P*-binding PX domain. Our data suggest that SGK3 activity is partially controlled by a pool of PtdIns(3)*P*, produced via phosphorylation of PtdIns by Vps34 and another pool of PtdIns(3)*P* that could be derived from dephosphorylation of PtdIns(3,4,5)*P*_3_ to PtdIns(3)*P* through the sequential actions of SHIP1/2 and INPP4B PtdIns phosphatases. VPS34-IN1 will be a useful probe for defining the roles that the class III PI3K plays. Moreover, analysing SGK3 phosphorylation and/or activity could be used as a downstream biomarker for Vps34 activity in the same way in which Akt phosphorylation is currently deployed to monitor class I PI3K pathway.

## MATERIALS AND METHODS

### Materials

Protein G–Sepharose was from GE Healthcare. [γ-^32^P]ATP was from PerkinElmer. Agarose-conjugated anti-FLAG M2 antibody, Triton X-100, EDTA, EGTA, sodium orthovanadate, sodium glycerophosphate, sodium fluoride, sodium pyrophosphate, 2-mercaptoethanol, sucrose, benzamidine, Tween 20, Tris/HCl, sodium chloride, magnesium acetate and doxycyclin were from Sigma. PMSF was from Melford. Tissue culture reagents, Novex 4–12% Bis-Tris gels and NuPAGE LDS sample buffer was from Invitrogen. Ampicillin was from Merck. P81 phosphocellulose paper was from Whatman. Methanol and chloroform were from VWR Chemicals. Inhibitors GDC-0941 (Axon Medchem), GSK2334470 (Tocris), AZD8055 (Selleck) and BKM120 (Chemie Tek) were purchased from the indicated suppliers. VPS34-IN1 (1-[{2-[(2-chloropyridin-4yl)amino]-4′-(cyclopropylmethyl)-[4,5′-bipyrimidin]-2′-yl}amino]-2-methyl-propan-2-ol) was synthesized as described in patent WO 2012085815 A1 [Cornella Taracido, I., Harrington, E.M., Honda, A. and Keaney, E. (2012) Preparation of bipyrimidinamine derivatives for use as Vps34 inhibitors; method for synthesis of this compound is described on page 73, Table 4, example 16a.VPS34-IN1 has a CAS registry number 1383716-33-3].

### General methods

Recombinant DNA procedures were performed using standard protocols. Mutagenesis was performed using the QuikChange site-directed mutagenesis (Stratagene) with KOD polymerase (Novagen). DNA constructs were purified from *Escherichia coli* DH5α using a Maxi prep kit (Qiagen) according to the manufacturer's instructions. Verification of the constructs was performed by the Sequencing Service (MRC Protein Phosphorylation Unit, College of Life Sciences, University of Dundee, U.K.; http://www.dnaseq.co.uk). DNA for bacterial protein expression was transformed into *E. coli* BL21-CodonPlus (DE3)-RIL cells (Stratagene). All recombinant proteins generated for the present study have an assigned [DU] number and are described on the reagents website (http:s://mrcppureagents.dundee.ac.uk/). Recombinant proteins used in the present study were as follows: Vps34/15 [DU8692], GST–TAPP1-(195–315) [DU17323], GST–Grp1-(241–399) [DU3464], GST–PLCδ-(1–178) [DU12981], 3×FLAG–SGK3-(1–162) wild-type [DU44877], 3×FLAG–SGK3-(1–162) R50A [DU44923] and 3×FLAG–SGK3-(1–162) R90A [DU44883].

### Cell culture, transfection and cell lysis

U20S cell line was kindly provided by John Rouse (MRC Protein Phosphorylation and Ubiquitylation Unit, University of Dundee, Dundee, U.K.). Cells were cultured in DMEM (Dulbecco's modified Eagle's medium) supplemented with 10% (v/v) FBS, 2 mM L-glutamine, 100 U/ml penicillin and 0.1 mg/ml streptomycin. cDNA for mouse Hrs (residues 147–223 ×2 joined by a linker) were cloned into a pBABE.puro vector. The construct was co-transfected into HEK (human embryonic kidney)-293FT cells with GAG/POL and VSV-G (vesicular stomatitis virus glycoprotein) expression plasmids (Clontech) for retrovirus production using Lipofectamine 2000 (Life Technologies) in accordance with the manufacturers’ instructions. The virus was harvested 48 h after transfection and applied to U20S cells in the presence of 10 μg/ml polybrene. Cells were selected with 10 μg/ml puromycin before single cell colony selection to identify low expression GFP–FYVE_Hrs_ clones. U2OS Flp/In cell line was kindly provided by John Rouse. Cells were cultured in McCoy medium supplemented with 10% (v/v) FBS, 2 mM L-glutamine, 100 U/ml penicillin and 0.1 mg/ml streptomycin. Stable cell lines expressing doxycycline-inducible proteins were generated using the Flp-In™ T-REx™ system (Invitrogen). Stable transfected clones were selected using 0.01 mM hygromycin in cell medium. Protein expression was induced by adding 0.01 mg/ml doxycycline in the medium for 24 h prior to cell lysis. Inhibitor treatment was carried out as described in the Figure legends. The cells were lysed in buffer containing 50 mM Tris/HCl (pH 7.5), 150 mM NaCl, 1 mM EDTA, 1 mM EGTA, 1 mM sodium orthovanadate, 10 mM sodium glycerophosphate, 50 mM sodium fluoride, 10 mM sodium pyrophosphate, 0.27 M sucrose, 0.1% 2-mercaptoethanol, 1 mM benzamidine and 0.1 mM PMSF. Lysates were clarified by centrifugation at 16000 ***g*** for 10 min at 4°C. Protein concentration was calculated using Bradford assay (Thermo Scientific). Immunoblotting and immunoprecipitation were performed using standard procedures. The signal was developed using the ECL Western Blotting Detection kit (GE Healthcare) on Hyperfilm ECL film (GE Healthcare).

### Antibodies

The following antibodies were raised in sheep, by the DSTT (Division of Signal Transduction Therapy) at the University of Dundee, and affinity-purified against the indicated antigens: anti-Akt1 (S695B, third bleed; raised against residues 466–480 of human Akt1: RPHFPQFSYSASGTA), anti-PRAS40 (proline-rich Akt substrate 40 kDa) (S115B, first bleed; raised against residues 238–256 of human PRAS40: DLPRPRLNTSDFQKLKRKY), anti-(phospho-PRAS40 Thr^246^) (S114B, second bleed; raised against residues 240–251 of human PRAS40: CRPRLNTpSDFQK), anti-NDRG1 (N-Myc downstream-regulated gene-1) (S276B third bleed; raised against full-length human NDRG1) and anti-SGK3 [S037D second bleed; raised against human SGK3 PX domain comprising residues 1–130 of SGK3]. Anti-phospho-Akt Ser^473^ (9271), anti-phospho-Akt Thr^308^ (4056), anti-phospho-NDRG1 Thr^346^ (5482), anti-GAPDH (2118), anti-phospho-4E-BP1 (eukaryotic initiation factor 4E-binding protein 1) Thr^37/46^ (9459), anti-phospho-4E-BP1 S65 (9451), anti-4E-BP1 (9452) and anti-phospho-SGK3 Thr^320^ (5642) antibodies were purchased from Cell Signaling Technology (note we found that this antibody did not recognize T-loop of SGK1 or SGK2). Anti-(phospho-SGK hydrophobic motif [Ser^486^ in SGK3]) antibody (sc16745) was from Santa Cruz Biotechnology and total SGK1/2 antibody was from Sigma (5188) as was the mouse anti-FLAG antibody. Secondary antibodies coupled to HRP (horseradish peroxidase) were obtained from Thermo Scientific.

### *In vitro* Vps34 PI3K assay

The PI3K activity of recombinant Vps34–Vps15 complex expressed in insect cells was assayed *in vitro* via a radioactive liposome kinase assay. Liposomes were formed by extrusion of crude liver PtdIns (Avanti Polar Lipids, 840042) through a 100 nm filter membrane and incubated with 50 ng of recombinant Vps34/15 for 30 min at 1000 rev./min and 4°C. Assay buffer was 20 mM Tris/HCl, pH 7.4, 67 mM NaCl, 10 mM MnCl_2_, 0.02% CHAPS and 1 mM DTT in the presence of 5 μM ATP and 3 μCi [γ-^32^P]ATP per reaction. Reactions were terminated by the addition of 500 μl of chloroform/methanol/HCl (100:200:3.5, by vol.) and subsequently 180 μl of chloroform and 300 μl of 0.1 M HCl to phase split reactions. The lower lipid-containing phase was retained and dried before the addition of chloroform. Reactions were spotted on to a Silica 60 TLC plate [activated with 1% potassium oxalate, 5 mM EDTA and 50% (v/v) methanol] and separated by a solvent comprising methanol/chloroform/water/ammonium bicarbonate (47:60:11.2:2, by vol.). Radioactive incorporation was analysed by Fujifilm Image reader FLA-2000 and quantified by AIDA image analysis, IC_50_ values were determined by non-linear regression using Prism software.

### AstraZeneca lipid kinase profiling

All compounds or DMSO for the PI4Kα, PI4Kβ, PIP5Kγ and PI3Kα biochemical, and IP1 (inositol phosphate) cell-based, assays were dispensed from source plates containing compounds at 10 mM in 100% (v/v) DMSO or 100% DMSO, directly into assay plates using an ECHO 555 Acoustic dispenser (Labcyte™).

PI4Kα and PI4Kβ were assayed using the ADP-Glo™ Kinase Assay Kit (Promega), in Greiner 384-well white low-volume plates in a 5 μl reaction volume consisting of 20 mM Bis-Tris propane (pH 7.5), 10 mM MgCl_2_, 0.5 mM EGTA, 1 mM DTT, 0.075 mM Triton X-100, 1.5% DMSO with or without an inhibitor at varying concentrations, D-*myo*-PtdIns substrate (Echelon Biosciences), ATP and purified enzyme. The PI4Kα assay was performed with 1 nM PI4KCA (Millipore), 72 μM ATP (KMappATP) and 25 μM PtdIns (KMappPI). The PI4Kβ assay was performed with 2 nM PI4KCB (SignalChem), 220 μM ATP (KMappATP) and 50 μM PtdIns (<KMappPI). The assay was allowed to proceed for 45 min at ambient temperature before stopping the reaction by the addition of 5 μl of ADP-Glo reagent. Plates were then covered and incubated for 40 min at ambient temperature. Kinase detection reagent (10 μl) was then added and the plates were incubated for 30 min before the luminescence signal was read with a PHERAstar plate reader (BMG Labtech).

PIP5Kγ assay was performed with the ADP-Glo™ Kinase Assay Kit (Promega) in Greiner 384-well white low-volume plates in a 5 μl reaction volume consisting of 20 mM Bis-Tris propane (pH 7.0), 10 mM MgCl_2_, 0.5 mM EGTA, 1 mM DTT, 0.024 mM Triton X-100, 1.5% DMSO with or without an inhibitor at varying concentrations, 14 μM D-*myo*-PIP (PtdIns 4-phosphate) substrate [<KMappPI(4)P] (Echelon Biosciences), 20 μM ATP (KMappATP) and PIP5K1C (expressed and purified by CRT, used at a 1:750 dilution). Assay incubations, additions and reads were performed as described for the PI4Kα and PI4Kβ ADP-Glo assays.

The PI3Kα assay was performed using the Kinase-Glo® Plus Luminescence Assay Kit (Promega) in Greiner 384-well white low-volume plates. PIK3CA (expressed and purified by AZ; 20 nM) in phosphorylation buffer consisting of 50 mM Tris/HCl (pH 7.4), 0.05% CHAPSO {3-[(3-cholamidopropyl)dimethylammonio]-2-hydroxy-1-propanesul-fonic acid}, 10 mM MgCl_2_ and 2.1 mM DTT was pre-incubated with or without an inhibitor at varying concentrations for 20 min, before adding 80 μM PtdIns(4,5)*P*_2_ substrate (Cayman Chemicals) and 8 μM (<KMappATP) ATP, to give a final assay volume of 6 μl [2% (v/v) DMSO assay final]. The assay was allowed to proceed for 80 min at ambient temperature, before the assay was stopped with the addition of 4 μl of kinase Glo® Plus reagent. Plates were then covered and incubated for 20 min before the luminescence signal was read with a PHERAstar plate reader (BMG Labtech).

The IP1 cell-based assay was performed using the HTRF IP-One Tb kit (CisBio) in Greiner 384-well tissue culture-treated white low-volume plates. NIH 3T3 cells, stably transfected using SV40 with the PDGFRβ receptor, were cultured in DMEM, 10% FBS, 1% Glutamax™ and 2 ng/ml puromycin. Cells were starved 24 h before use in DMEM, 1% charcoal-stripped FBS and 1% Glutamax™. After harvesting, cells were resuspended at a final concentration of 1.25×10^6^ cells/ml in IP1 cell stimulation buffer, and 8 μl was dispensed into each well of the assay plate pre-dosed with compound or DMSO. After incubation for 30 min at 37°C, cells were then stimulated with 150 nM PDGF (Sigma–Aldrich) dispensed using an ECHO 555. A standard curve IP1 calibration plate was prepared according to the manufacturer's instructions. After incubation for 30 min at 37°C, cells were lysed with the addition of 3 μl of lysis buffer with IP-One d2 (1:20 dilution), and 3 μl of lysis buffer with anti-IP-One Tb cryptate (1:20 dilution). Plates were covered and incubated for 2 h at ambient temperature, before a HTRF read at 615 nm and 665 nm on an EnVision plate reader (PerkinElmer). Assay values were then normalized to the IP1 calibration curve.

### Immunoprecipitation and assay of SGK3 and SGK2

*In vitro* kinase activity of SGK3 and SGK2 was assayed by measuring [γ-^32^P]ATP incorporation into Crosstide substrate peptide [GRPRTSSFAEGKK]. SGK3 with a C-terminal FLAG-tag was immunoprecipitated from doxycycline-induced U2OS Flp/In cell lines. Immunoprecipitates were washed in sequence with lysis buffer containing high salt concentration (500 mM NaCl), lysis buffer and buffer A (50 mM Tris/HCl, pH 7.5, and 0.1 mM EGTA). Reactions were carried in 40 μl of total volume containing 0.1 mM [γ-^32^P]ATP (400–1000 c.p.m./pmol), 10 mM magnesium acetate and 30 μM Crosstide peptide. Reactions were terminated by adding 10 μl of 0.1 mM EDTA and spotting 40 μl of the reaction mixture on P81 paper which were immediately immersed into 50 mM orthophosphoric acid. Papers were washed several times in 50 mM orthophosphoric acid, rinsed in acetone and air dried. Radioactivity was quantified by Cerenkov counting. One unit of enzyme activity was defined as the amount of enzyme that catalyses incorporation of 1 nmol of [γ-^32^P]ATP into the substrate over 1 min.

### Immunofluorescence and time-lapse microscopy

Cells were cultured on glass coverslips and processed for immunocytochemistry using standard protocols. For experiments investigating the subcellular distribution of SGK3 C-terminally tagged GFP wild-type and mutant proteins and GFP–PH_Akt_ cells were fixed with 4% paraformaldehyde for 15 min at room temperature and processed for imaging without permeabilization. For experiments quantifying co-localization of SGK3–GFP with EEA1 (early endosome antigen 1) and localization GFP–FYVE_Hrs_ in response to inhibitor treatment, cells were permeabilized via one freeze–thaw cycle in liquid nitrogen and subsequently fixed. The following primary antibodies were used: rabbit anti-EEA1 (1:500 dilution; Life Technologies) and mouse anti-GFP (1:1000 dilution; Life Technologies) antibodies. Fluorophore-conjugated secondary antibodies (Alexa Fluor® 594 and Alexa Fluor® 488) were obtained from Life Technologies. Imaging was conducted using a Nikon Eclipse Ti-S microscope (×40 objective) or a Zeiss LSM710 laser scanning confocal microscope [×63 Plan-Apochromat oil immersion objective; 1.40 NA (numerical aperture)] as described in the Figure legends. Time-lapse microscopy was carried out on the same microscope using a heated incubator, heated stage plate and CO_2_ controller (Pecon) to maintain the cells.

Images were acquired every 0.5 min up to 60 min. Inhibitors were added after acquiring two images, except for VPS34-IN1 that was added after the first image taken. Post-acquisition, image analysis was conducted with Volocity 6.3 (PerkinElmer).

### Lipid overlay assay

The assay was performed as described previously [33]. Briefly, 500 pmol of each lipid [Ptdins, PtdIns(3)*P*, PtdIns(4)*P*, PtdIns(5)*P*, PtdIns(3,4)*P*_2_, PtdIns(4,5)*P*_2_, Ptdins(3,5)*P*_2_ and PtdIns(3,4,5)*P*_3_] were spotted on to Hybond-C membrane (GE Healthcare) and let dry for 1 h. The membrane was blocked in blocking buffer (50 mM Tris/HCl, pH 7.5, 150 mM NaCl, 0.1% Tween-20 and 2 mg/ml fatty acid-free BSA) for 1 h. The membrane was incubated overnight at 4°C in a 10 nM solution of each recombinant protein. After washing in TBST buffer (50 mM Tris/HCl, pH 7.5, 150 mM NaCl, 0.1% Tween 20), the signal was detected by using HRP-conjugated anti-FLAG or HRP-conjugated anti-GST antibodies (Roche) at a 1:3000 dilution. All lipids were purchased from Echelon and dissolved in 1:1 solution of methanol and chloroform.

### ITC (isothermal titration calorimetry)

ITC experiments were carried out on a Microcal VP-ITC. A series of injections of peptide were made into an isolated chamber containing the protein at a constant temperature of 30°C. Heat changes within the cell were monitored during each injection of peptide and recorded as the total heat change per second over time. A binding isotherm was then fitted to data and expressed as the heat change per mol of peptide against the peptide/protein ratio. Injectant was PtdIns and PtdIns(3)*P* (Echelon) dissolved in buffer (50 mM Hepes, pH 7.4, and 150 mM NaCl). The chamber contained recombinant 3×FLAG–SGK3-(1–162), 3×FLAG–SGK3-(1–162) R50A and 3×FLAG–SGK3 R90A in a buffer (50 mM Hepes, pH 7.4, and 150 mM NaCl). During each experiment, 40×6 μl doses of peptide were injected into the chamber of protein, which was stirred constantly at 300 rev./min. Each injection was followed by a 3 min period to ensure equilibration of the solution. All experiments were repeated and where possible using different concentrations of separately prepared protein. Data were analysed using Origin 7.0 with the Microcal software patch installed. Each experimental condition had a blank run with protein in the chamber replaced with buffer. These data were then subtracted from the run with protein present to take into account any energy of dilution or metal/buffer reaction. A binding isotherm was then fitted to the data using a least squares calculation to yield a χ^2^ value. The model that produced the lowest χ^2^ value was taken as the best fit.

### Statistical analysis

All experiments in the present study were performed at least twice and similar results were obtained. Data were analysed using one-way ANOVA followed by Bonferroni's post-hoc test (*P*<0.05) using Prism software. The error bars indicate S.D.

## RESULTS

### VPS34-IN1 is a potent and selective inhibitor of Vps34 class III PI3K

**Figure 1:**
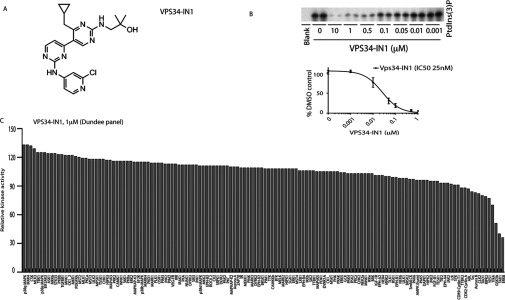
VPS34-IN1, a selective inhibitor of Vps34 kinase (**A**) Chemical structure of the VPS34-IN1. (**B**) Insect cell expressed recombinant human Vps34–Vps15 complex was assayed by measuring phosphorylation of PtdIns in a ^32^P-radioactive kinase assay in the absence or presence of the indicated concentrations of VPS34-IN1. Reactions were chromatographed on a Silica 60 TLC plate and ^32^P-radioactivity associated with the spot comprising PtdIns(3)*P* was visualized (top panel) and quantified (bottom panel) by phosphoimager analysis on a Fujifilm Image reader FLA-2000 employing the AIDA image analysis software. Data are shown as the mean kinase activity±S.D. for three independent experiments, relative to DMSO-treated sample. The IC_50_ histogram was generated using Prism Software with non-linear regression analysis. (**C**) Protein kinase profiling of the VPS34-IN1 at a single concentration of 1 μM carried out against the Dundee panel of 140 protein kinases at the International Centre for Protein Kinase Profiling. Results for each kinase are presented as the mean kinase activity±S.D. for an assay undertaken in triplicate relative to a control kinase assay in which the inhibitor was omitted. Abbreviations and assay conditions used for each kinase are defined at http://www.kinase-screen.mrc.ac.uk.

The structure of the bipyrimidinamine VPS34-IN1 compound is shown in Figure 1(A). This compound was originally reported in a patent (WO 2012085815 A1). We found that VPS34-IN1 inhibited phosphorylation of PtdIns by recombinant insect cell expressed Vps34–Vps15 complex with an IC_50_ of ~25 nM employing both an in-house assay (Figure 1B) or using an external assay undertaken by Life Technologies (Supplementary Figure S1).

To evaluate the selectivity of VPS34-IN1, we studied the effect that this compound had on the activity of two protein kinase panels namely the Dundee panel (140 kinases) (Figure 1C, and Supplementary Table S1) and the ProQinase panel (300 kinases) (Supplementary Figure S2). These data revealed that VPS34-IN1 was remarkably selective and at a concentration of 1 μM, did not significantly inhibit the activity of any of the protein kinases assessed.

**Figure 2:**
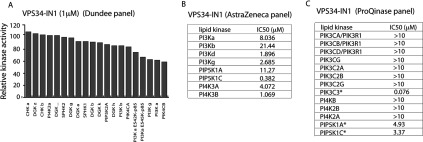
VPS34-IN1 selectively inhibit class III PI3K (**A**) Lipid kinase profiling of the VPS34-IN1 at 1 μM was carried out against a panel of 19 lipid kinases at the International Centre for Protein Kinase Profiling. (**B**) Summary of the measurements of VPS34-IN1 for the indicated kinases and IP1 levels in cells (see the Materials and methods section for assay conditions). (**C**) Lipid kinase profiling of VPS34-IN1 inhibitor was carried out against ProQinase panel of 13 lipid kinases. Summary of IC_50_ measurements for each kinase is presented in the Table. Abbreviations used for each kinase and assay conditions used are defined at http://www.proqinase.com. Dose–response curves for data marked with an asterisk are presented in Supplementary Figure S3.

We next evaluated VPS34-IN1 against three different lipid kinase profiling panels namely the Dundee panel (Figure 2A, 19 lipid kinases, includes class I PI3Ks), the AstraZeneca panel (Figure 2B, eight lipid kinases, includes class I) and ProQuinase panel (Figure 2C and Supplementary Figure S3, 13 lipid kinases, includes class I and class II PI3Ks). This revealed that VPS34-IN1 did not significantly inhibit any of the lipid kinases tested including class I (p110α, p110β p110γ and p110δ) and all three members of the class II PI3Ks (PI3KC2α, PI3KC2β and PI3KC2γ) enzymes.

### VPS34-IN1 inhibits PtdIns(3)*P* levels at endosomes in a dose-dependent manner

**Figure 3:**
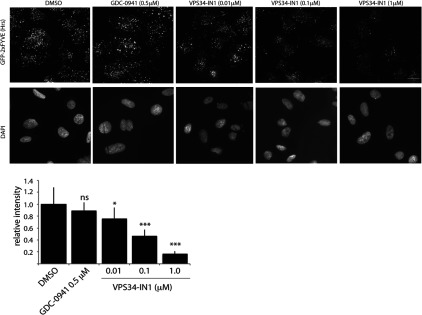
VPS34-IN1 reduces GFP–2×FYVE_Hrs_ probe localization on endosomes in a dose-dependent manner U2OS cell line stably expressing GFP–2×FYVE_Hrs_ was treated with indicated concentration of VPS34-IN1 inhibitor for 1 h. The cells were permeabilized by freeze–thaw in liquid nitrogen and fixed in 4% paraformaldehyde. The GFP signal was enhanced by using mouse anti-GFP primary and anti-mouse Alexa Fluor® 488 secondary antibody. The top panel shows representative cell images of GFP–2×FYVE_Hrs_ localization and the bottom panel shows corresponding DAPI staining for each condition. The histogram displays average cell fluorescence±S.D. compared with DMSO-treated control. Similar result was obtained in at least one other experiment. The average cell fluorescence was calculated by dividing total fluorescence of each field by the number of the cells in the field. For each condition, ten random fields were chosen containing 20–25 cells/field. Images were taken using Nikon Eclipse Ti-S microscope using ×40 objective. Analysis was performed using NIS-Elements BR3.1 program. Scale bar, 20 μm. *P≤0.05, ***P≤0.001. ns, not significant.

The major pool of cellular PtdIns(3)*P* generated in cells by Vps34 accumulates on the surface of endosome membranes, which can be visualized using a GFP-tagged probe fused to a tandem repeat of the FYVE domain of the endocytic pathway Hrs protein [31]. Consistent with previous work, we observed that GFP–2×FYVE_Hrs_ when stably expressed in U2OS osteosarcoma cells localized to discrete punctate cytoplasmic structures characteristic of endosomes (Figure 3). Treatment with Vps34-IN1 for 1 h induced a marked dose-dependent suppression of endosomal localization of GFP–2×FYVE_Hrs_ with ~80% loss observed at 1.0 μM and ~50% loss at 0.1 μM (Figure 3). We did not observe significant reduction in binding when employing PI3K class I inhibitor GDC-0941.

### VPS34-IN1 rapidly reduces PtdIns(3)*P* levels at endosomes

**Figure 4:**
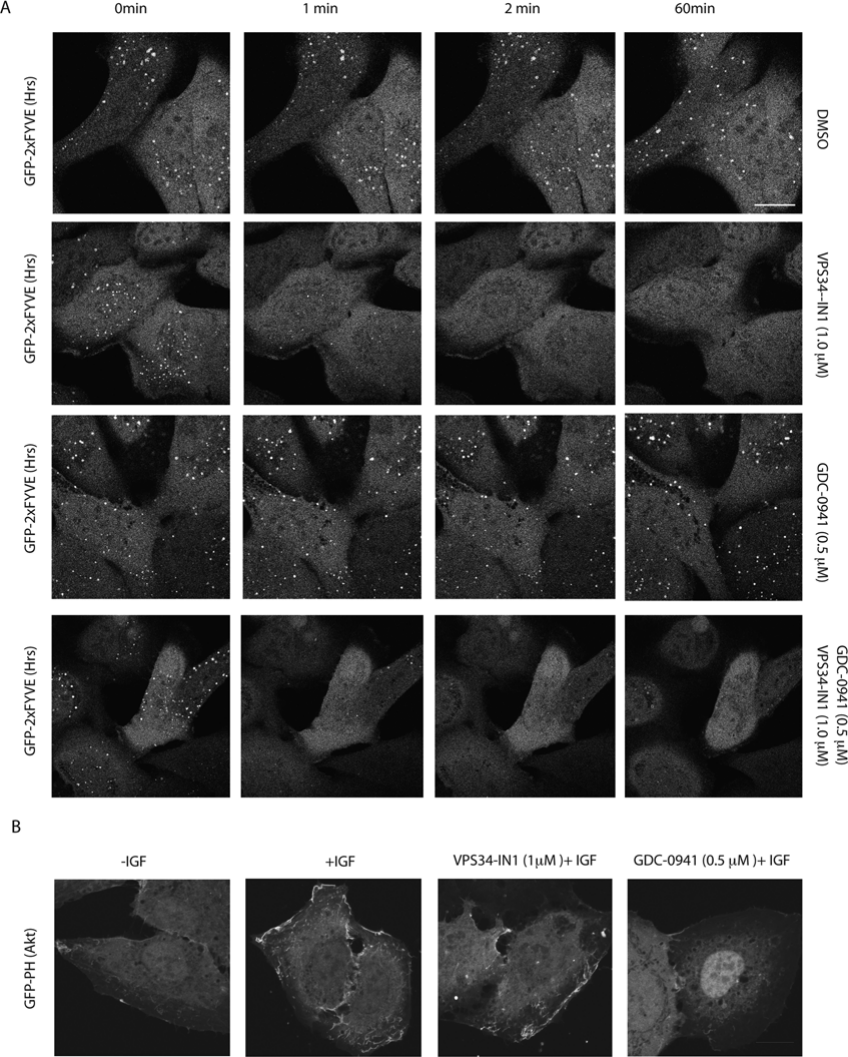
VPS34-IN1 rapidly reduces GFP–2×FYVE_Hrs_ probe localization on endosomes (**A**) The U2OS cell line stably expressing GFP–2×FYVE_Hrs_ was recorded for approximately 1 min, before adding either no inhibitor (top panel), 1 μM VPS34-IN1 (second panel), 0.5 μM GDC-0941 (third panel) or a combination of 1 μM VPS34-IN1 and 0.5 μM GDC-0941 (bottom panel). Images were taken starting at 1.5 min from the time that the inhibitor was added (the first time point we could reliably measure) and subsequently at 0.5 min intervals up to a period of 1 h. Time-lapse microscopy was performed on Zeiss 710 microscope using ×63 objective. Similar results were obtained in at least two separate experiments. Scale bar, 20 μm. (**B**) As in (**A**) except the U2OS cell line stably expressing GFP–PH_Akt1_ was starved of serum overnight and treated with either no inhibitor (left panel), 1 μM VPS34-IN1 (middle panel) or 0.5 μM GDC-0941 (right panel) for 1 h before stimulation with IGF (100 ng/ml for 15 min). The cells were fixed with 4% paraformaldehyde and images were taken using Zeiss 710 microscope at ×63 objective. Representative images are shown and similar results were obtained in two experiments. Scale bar, 20 μm.

We also undertook a live imaging analysis monitoring the effect of 1 μM VPS34-IN1 on localization GFP–2×FYVE_Hrs_ with time. The data demonstrated that the effect of VPS34-IN1 in dispersing punctate endosomal localization of GFP–2×FYVE_Hrs_ was rapid with significant effects observed within 1 min (the earliest time-point that can be reliably analysed) and near maximal effects within 2 min (Figure 4A). Low levels of endosomal localization of GFP–2×FYVE_Hrs_ were maintained for up to 60 min, the longest time point we analysed (Figure 4). As expected treatment of U2OS cells with 0.5 μM GDC-0941, a class I PI3K inhibitor that does not inhibit Vps34 [34], had no effect on endosomal localization of GFP–2×FYVE_Hrs_ even after 60 min treatment (Figure 4A). Combination of VPS34-IN1 and GDC-0941 dispersed endosomal localization of GFP–2×FYVE_Hrs_ similarly to VPS34-IN1 alone (Figure 4A). It should be noted that low levels of residual PtdIns(3)*P* were still observed in cells treated with both VPS34-IN1 and GDC-0941, which could represent PtdIns(3)*P* generated through class II PI3K which was not inhibited by either inhibitor. We also found that 1 μM VPS-34-IN1 had no impact on the ability of IGF1 (insulin-like growth factor 1) to induce plasma membrane recruitment of a GFP probe encompassing the isolated PtdIns(3,4,5)*P*_3_/PtdIns(3,4)*P*_2_ binding PH domain of Akt1 (GFP–PH_Akt1_) (Figure 4B). In parallel experiments the class I PI3K GDC-0941 inhibitor blocked IGF1-induced translocation of the GFP–PH_Akt1_ probe to the plasma membrane (Figure 4B).

### PtdIns(3)*P* binding to PX domain localizes SGK3 to endosomes

**Figure 5:**
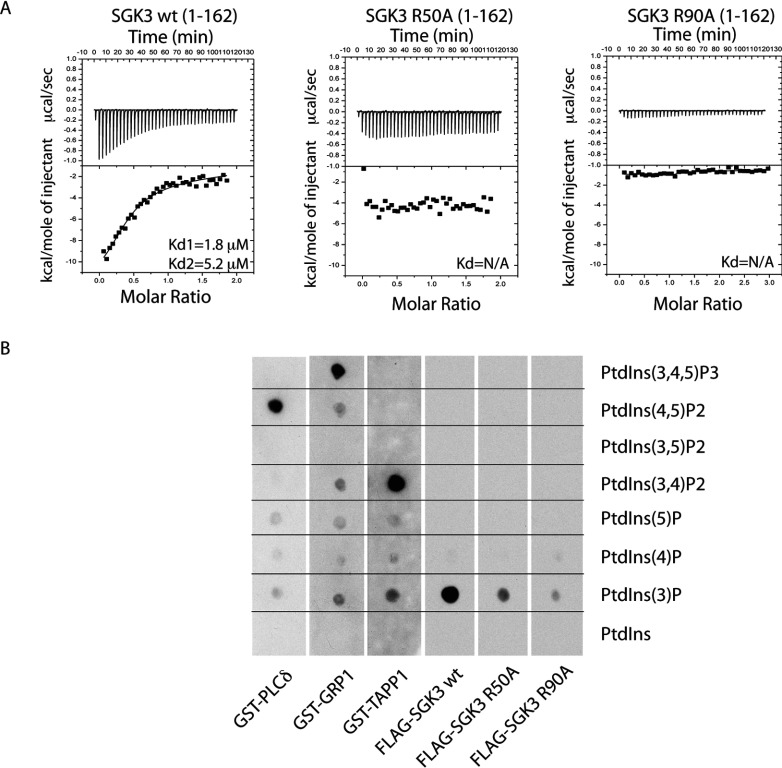
SGK3 binds to PtdIns(3)*P* via its PX domain (**A**) ITC was performed by gradually titrating 0.5 mM PtdIns(3)*P* into the reaction chamber containing wild-type 3×FLAG–SGK3-(1–162) PX domain (left panel) or mutant 3×FLAG–SGK3-(1–162) R50A PX domain (middle panel) and 3×FLAG–SGK3-(1–162) R90A PX domain (right panel). The top panel illustrates enthalpic heat released during titration at 30°C and the bottom panel presents integrated binding isotherms and the best fit curves. *K*_d_ are indicated on the graphs. The measurement was performed on a VP-ITC MicroCalorimeter MicroCal machine. *K*_d_ were calculated using Origin 7.0 with ITC data analysis disc program. (**B**) The ability of the indicated GST fusion proteins to bind various phosphoinositides was analysed. The indicated phosphoinositides (500 pmol) were spotted on to nitrocellulose membranes, which were then incubated with 10 nM of the wild-type and indicated mutants of 3×FLAG–SGK3-(1–162) PX domain or GST-fusion PH domains [PLCδ-(1–178), GRP1-(241–399), TAPP1-(195–315) proteins]. The membranes were washed and the PX domain protein bound to the membrane by virtue of its interaction with lipid was detected using by using HRP-conjugated anti-FLAG antibodies (SGK3 PX domain) or anti-GST (PH domains).

We speculated that one downstream target of Vps34 might comprise the serum- and glucocorticoid-induced protein kinase SGK3, which is unique among kinases in that it possesses an N-terminal PX domain that binds specifically to PtdIns(3)*P* [35,36]. Using isothermal calorimetry, we confirmed previous work [35,36] that the isolated PX domain of SGK3 (residues 1–162, PX^SGK3^) interacted with PtdIns(3)*P* (*K*_d1_ of 1.8 μM and *K*_d2_ of 5.2 μM), but not with unphosphorylated PtdIns (results not shown). Molecular modelling of the non-complexed crystal structure of PX^SGK3^ suggested that the conserved Arg^50^ and Arg^90^ residues located within the PX domain might form ionic interactions with the 3′-phosphate of PtdIns(3)*P* [37]. We therefore mutated Arg^50^ or Arg^90^ to alanine and found that these mutations abolished binding of PX^SGK3^ to PtdIns(3)*P* in either ITC (Figure 5A) or protein lipid overlay binding [33] assays (Figure 5B). We also corroborated previous work [35,36], that PX^SGK3^ binds specifically to PtdIns(3)*P* and not to a range of other phosphoinositides tested [PtdIns, PtdIns(4)*P*, PtdIns(5)*P*, PtdIns(4,5)*P*_2_, PtdIns(3,4)*P*_2_ or PtdIns(3,4,5)*P*_3_] under conditions which Grp1 PH domain interacted with PtdIns(3,4,5)*P*_3_, the TAPP1 PH domain interacted with PtdIns(3,4)*P*_2_ and the phospholipase Cδ PH domain bound PtdIns(4,5)*P*_2_ [33] (Figure 5B).

### PtdIns(3)*P* binding localizes SGK3 to early endosomes

**Figure 6:**
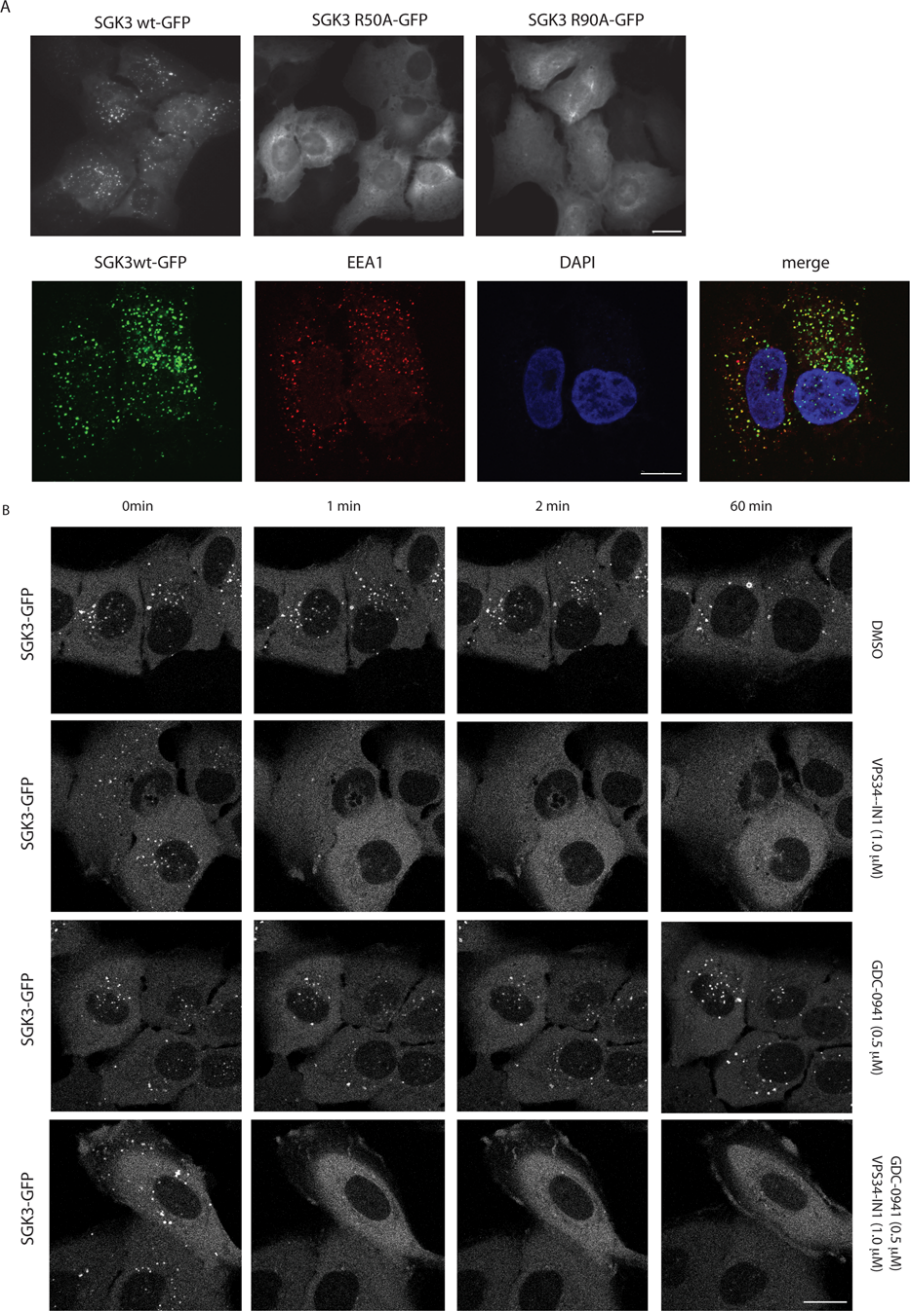
PtdIns(3)*P* binding localizes SGK3 to endosomes (**A**) U2OS stably expressing wild-type or indicated mutant of full-length SGK3 with a C-terminal GFP-tagged were fixed with 4% (v/v) paraformaldehyde and GFP distribution in cell was visualized. Images were taken using a Nicon Eclipse Ti-S microscope with ×40 objective (upper panel). SGK3 co-localization with early endosomal marker EEA1 marker was visualized using rabbit anti-EEA1 primary and anti-rabbit Alexa Fluor® 594 secondary antibody (lower panel). Pictures were taken with Zeiss 710 microscope using ×100 objective. Mander's correlation coefficient in 112 cells was calculated using the Volocity 6.3 program. Scale bar, 20 μm. (**B**) The U2OS cell line stably expressing full-length SGK3 with an C-terminal GFP-tagged were recorded for approximately 1 min, before adding either no inhibitor (top panel), 1 μM VPS34-IN1 (second panel), 0.5 μM GDC-0941 (third panel) or a combination of 1 μM VPS34-IN1 and 0.5 μM GDC-0941 (bottom panel). Images were taken starting at 1.5 min from the time that the inhibitor was added (the first time point we could reliably measure) and subsequently at 0.5 min intervals up to a period of 1 h. Time-lapse microscopy was performed on Zeiss 710 microscope using ×63 objective. Representative images are shown and similar results were obtained in two experiments. Scale bar, 20 μm.

We next analysed the cellular location in U2OS cells of full-length wild-type SGK3 possessing a C-terminal GFP tag. This revealed clear-cut punctate endosomal localization of wild-type SGK3 that overlapped with localization of the early endosomal EEA1 marker [38] (Figure 6A). In contrast, the non-PtdIns(3)*P*-binding SGK3(R50A) or SGK3(R90A) mutants were diffusely dispersed throughout the cytosol (Figure 6A). Using time-lapse microscopy we also observed that treatment of cells with 1 μM VPS34-IN1 resulted in the rapid dispersal of SGK3 from the endosomes within 1 min. In contrast, treatment with the class I PI3K inhibitor GDC-0941 (0.5 μM) did not change significantly the SGK3 localization on endosomes (Figure 6B). Dual treatment with VPS34-IN1 and GDC-0941 inhibitors revealed that the effect of inhibitors on PtdIns(3)*P* production was as rapid as with VPS34-IN1 alone resulting in reduced SGK3 punctate appearance.

### PtdIns(3)*P* binding controls SGK3 activity and hydrophobic motif phosphorylation

**Figure 7:**
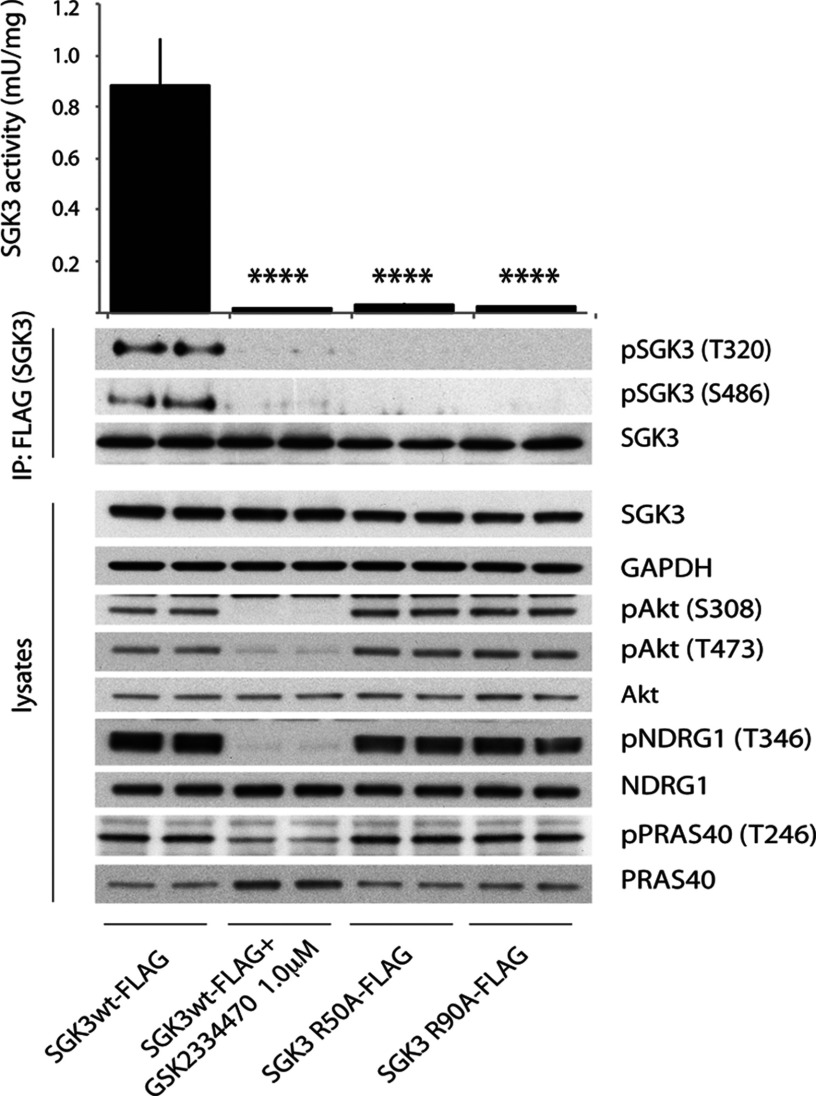
SGK3 phosphorylation and kinase activity is controlled by its ability to bind PtdIns(3)*P* (**A**) U2OS stably expressing wild-type or indicated mutant of full-length SGK3 with a C-terminal FLAG-tag were treated in the absence or presence of the indicated PDK1 inhibitor (1 μM GSK2334470) for 1 h. The cells were lysed and SGK3 immunoprecipitated with anti-FLAG antibody. The immunoprecipitates were subjected to immunoblot analysis with the indicated antibodies (lower panel) after being assayed for SGK3 kinase activity by measuring phosphorylation of the Crosstide substrate peptide in the presence of 0.1 mM [γ-^32^P]ATP in a 30 min reaction (upper panel). Kinase reactions are presented as means±S.D. for triplicate reaction. The similar result was obtained in at two separate experiments. ****P≤0.0001.

SGK3, similar to many other AGC kinases including Akt isoforms, is activated following phosphorylation of its catalytic domain T-loop motif (Thr^320^) by PDK1 and its non-catalytic C-terminal hydrophobic motif (Ser^486^) by mTOR [35,39,40]. Previous work with SGK1 has shown that mTORC2-mediated phosphorylation of the hydrophobic motif markedly enhances phosphorylation of the T-loop residue by creating a binding motif for PDK1 [40–42]. Consistent with previous work, we observed that full-length wild-type SGK3 when stably expressed in U2OS cells displayed considerable kinase activity towards the Crosstide peptide (assessed after immunoprecipitate kinase assay) and was also significantly phosphorylated at its T-loop and hydrophobic motifs (assessed employing phosphospecific antibodies recognizing these sites) (Figure 7). Strikingly, the non-PtdIns(3)*P*-binding SGK3(R50A) or SGK3(R90A) mutants displayed only low kinase activity and T-loop and hydrophobic motif phosphorylation, suggesting PtdIns(3)*P* binding to the PX domain plays a critical role in enabling SGK3 to become phosphorylated at its T-loop and hydrophobic motif and therefore become activated (Figure 7). As expected, treatment of cells with a selective PDK1 inhibitor (GSK2334470) [43] suppressed SGK3 activity, as well as T-loop and hydrophobic motif phosphorylation, to near basal levels, similar to those observed following mutation of the PX domain (Figure 7).

### Class I and class III PI3K inhibitors rapidly suppress SGK3 activity and T-loop/hydrophobic motif phosphorylation

**Figure 8:**
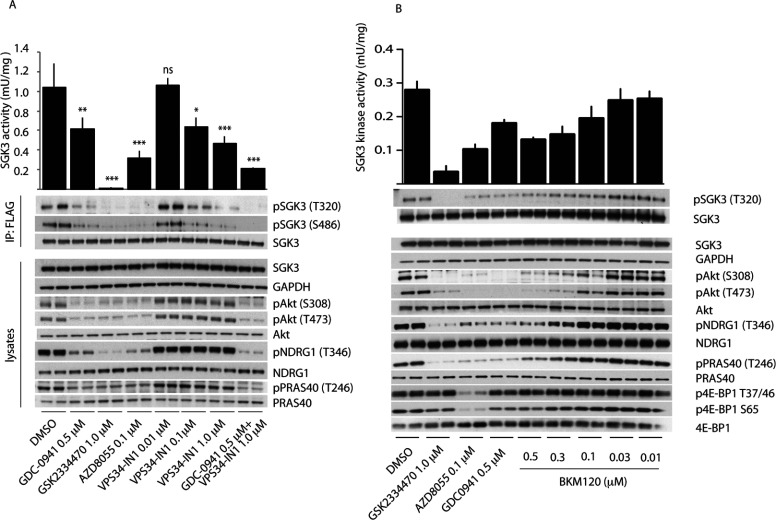
Evidence that SGK3 phosphorylation and kinase activity is controlled by Vps34 and class I PI3K (**A**) U2OS stably expressing wild-type or indicated mutant of full-length SGK3 with a C-terminal FLAG-tag were treated in the absence or presence of the indicated concentrations of VPS34-IN1 inhibitor or class I PI3K inhibitor (0.5 μM GDC-0941 [34]) PDK1 inhibitor (1 μM GSK2334470 [43]), mTOR inhibitor (0.1 μM AZD8055 [45]) for 1 h. The cells were lysed and SGK3 immunoprecipitated with anti-FLAG antibody. The immunoprecipitates were subjected to immunoblot analysis with the indicated antibodies (lower panel) after being assayed for SGK3 kinase activity by measuring phosphorylation of the Crosstide substrate peptide in the presence of 0.1 mM [γ-^32^P]ATP in a 30 min reaction (upper panel). Kinase reactions are presented as means±S.D. for triplicate reaction. (**B** and **C**) As in (**A**) except cells were treated with the indicated doses of the BKM120 class I PI3K inhibitor. Similar results were obtained in at least two separate experiments for all data shown in this Figure. ***P≤0.001, **P≤0.01 and *P≤0.05. ns, not significant.

We next treated U2OS cells stably expressing SGK3 with increasing doses of VPS34-IN1 for 1 h and tested how this affected SGK3 activity and T-loop phosphorylation. This revealed that VPS34-IN1 induced a dose-dependent reduction in SGK3 activity that was maximally lowered by ~60% at 1 μM and ~40% at 0.1 μM (Figure 8A). The suppression of SGK3 activity induced by VPS34-IN1 treatment was accompanied by a commensurate decrease in T-loop and hydrophobic motif phosphorylation (Figure 8A).

We also observed that addition of the selective class I PI3K inhibitor GDC-0941 induced a ~40% inhibition of SGK3 activity accompanied by a similar reduction in T-loop and hydrophobic motif phosphorylation (Figure 8A). Moreover, a structurally distinct selective class I PI3K inhibitor that is in clinical trials termed BKM120 [44] induced a comparable ~40% inhibition of SGK3 (Figure 8B). Consistent with GDC-0941 (Figure 8A) and BKM120 (Figure 8B) inhibiting a class I PtdIns kinase, they suppressed the Akt T-loop (Thr^308^) and hydrophobic motif (Ser^473^) phosphorylation, as well as Akt substrate PRAS40 phosphorylation (Thr^246^), to near basal levels. At the doses used neither GDC-0941 nor BKM120 was judged to significantly suppress mTOR activity based on the lack of effect that these compounds had on the phosphorylation of the 4EBP1 at sites phosphorylated by mTORC1 (Thr^37^, Thr^46^ and Ser^65^) (Figure 8B). Treatment of cells with the AZD8055 [45] mTOR inhibitor in parallel experiments led to marked dephosphorylation of 4E-BP1 at residues phosphorylated by mTOR (Figure 8B).

Combining 1.0 μM VPS34-IN1 and 0.5 μM GDC-0941 inhibitors reduced SGK3 activity and phosphorylation by over 80% to a similar extent as observed with treatment with AZD8055 mTOR inhibitor (Figure 8A).

**Figure 9:**
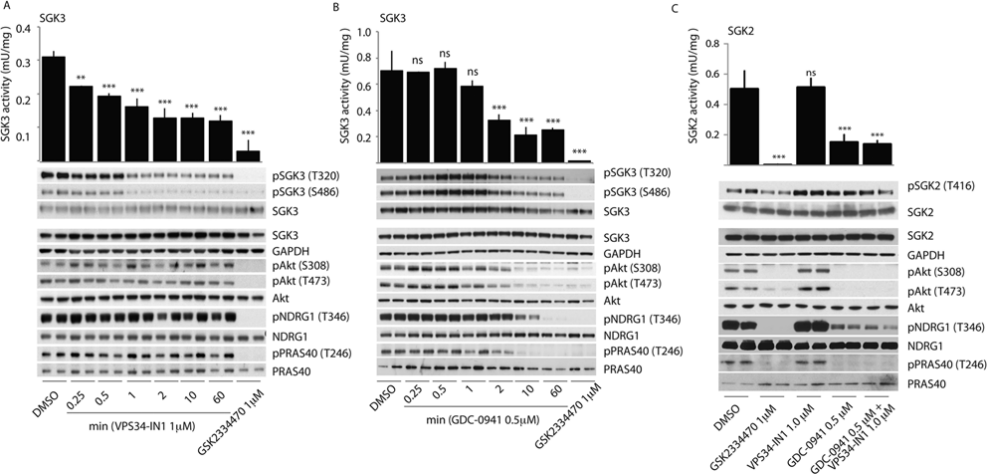
Class I and class III PI3K inhibitors rapidly inactivate SGK3 and evidence that SGK2 is not regulated by Vps34 (**A** and **B**) U2OS stably expressing wild-type full-length SGK3 with a C-terminal FLAG-tag were treated in the absence or presence of the 1 μM VPS34-IN1 (**A**) or 0.5 μM GDC-0941 (**B**) for the indicated times. The cells were lysed and SGK3 immunoprecipitated with anti-FLAG antibody. The immunoprecipitates were subjected to immunoblot analysis with the indicated antibodies (top panel) after being assayed for SGK3 kinase activity by measuring phosphorylation of the Crosstide substrate peptide in the presence of 0.1 mM [γ-^32^P]ATP in a 30 min reaction (bottom panel). Kinase reactions are presented as means±S.D. for triplicate reaction. (**C**) As above except U2OS stably expressing wild-type full-length SGK2 with a C-terminal FLAG-tag were treated in the absence or presence of the indicated inhibitors for 1 h. Similar results was obtained in at least two separate experiments for all data shown in this Figure. ***P≤0.001, **P≤0.01 and *P≤0.05. ns, not significant.

A time course analysis revealed that the effect of 1 μM VPS34-IN1 on SGK3 activity and phosphorylation was extremely rapid and activity reduced by 50% within 15 s and maximally suppressed within 1 min (Figure 9A). In contrast, the effect of GDC-0941 on SGK3 activity and phosphorylation was slower with little effect observed at a 1 min time point, but activity declining significantly thereafter with near maximal inhibition observed after 2 min of GDC-0941 treatment (Figure 9B).

Consistent with VPS34-IN1 not supressing class I PI3Ks, we observed that the inhibitor had no effect on Akt phosphorylation at Ser^473^ and Thr^308^ or phosphorylation of the Akt substrate PRAS40 (Figure 8A). We also monitored NDRG1 phosphorylation at Thr^346^ a site that can be phosphorylated by SGK or Akt isoforms [46]. In U2OS cells, as observed in some other cell types [46], it seems that phosphorylation of NDRG1 is mediated mainly through Akt as its phosphorylation is significantly suppressed by GDC-0941, but only affected moderately by VPS34-IN1 (Figure 8). Moreover, in agreement with the notion that the ability of SGK3 to be inhibited by VPS34-IN1 is dependent on its PtdIns(3)*P*-binding PX domain, we observed that VPS34-IN1 did not inhibit the activity of SGK2 isoform that does not possess a PX domain (Figure 9C).

## DISCUSSION

VPS34-IN1 is the first highly selective cell permeable Vps34 inhibitor to be reported. Previously utilized Vps34 inhibitors have included wortmannin [47], LY294002 [48], 3-methyladenine [47], KU55933 [49,50] and Gö6976 [50] that are not selective inhibitors. Wortmannin and LY294002 when used at concentrations that inhibit Vps34 also suppress class I PI3Ks and several kinases that belong to the PI3K-related kinase (mTOR and DNA-dependent protein kinase) in addition to many other protein and lipid kinases [51–53]. KU55933 potently inhibits the DNA damage repair ATM protein kinase and to our knowledge has not been thoroughly profiled against a panel of lipid kinases including class I and class II PI3Ks so the off-target effects of this compound are unknown. The protein kinase C inhibitor Gö6976 is poorly selective inhibiting many protein kinases (http://www.kinase-screen.mrc.ac.uk/screening-compounds/341062). To our knowledge extensive protein and lipid kinase profiling has not been undertaken for 3-methyladenine, but in our judgment this simple adenine derivative is unlikely to comprise a selective Vps34 inhibitor. In contrast, the selectivity data for VPS34-IN1 indicates that it is a remarkably selective Vps34 inhibitor. We have profiled VPS34-IN1 against 340 unique protein kinases (Figure 1 and Supplementary Figure S2) and 25 lipid kinases that include all four members of the class I PI3K and three members of the class II PI3Ks (Figure 2, and Supplementary Figures S1 and S3). We have found that, other than Vps34, no kinase is markedly inhibited at a concentration of 1 μM, which is 40-fold higher than the IC_50_ value that VPS34-IN1 inhibits Vps34 and the highest concentration of compound we have used in cells. It is reassuring that VPS34-IN1 does not inhibit any of the three members of the class II PI3Ks, as these poorly understood enzymes, which are similar to Vps34, also generate PtdIns(3)*P*. This provides confidence that the impact that VPS34-IN1 has on endosomal PtdIns(3)*P* levels and SGK3 activity *in vivo* is indeed due to inhibition of PtdIns(3)*P* generated through the action of Vps34.

The evidence that VPS34-IN1 suppresses Vps34 activity *in vivo* is supported by the observation that treatment of cells with VPS34-IN1 induces a strikingly rapid dose-dependent dispersal of the localization of the PtdIns(3)*P*-binding GFP–2×FYVE_Hrs_ from endosomal membranes (Figures 3, 4 and 6), where PtdIns(3)*P* is produced by Vps34 resides [31,54]. Consistent with the *in vitro* IC_50_ of VPS34-IN1 for Vps34 being 25 nM (Figure 1B and Supplementary Figure S1), treatment of cells with 0.01 μM VPS34-IN1 induced a weak effect on GFP–2×FYVE_Hrs_ localization, whereas 0.1 μM and 1 μM VPS34-IN1 induced ~50% and ~80% respectively dispersal of GFP–2×FYVE_Hrs_ endosomal localization (Figure 7). The effect of VPS34-IN1 in dispersing the endosome-localized GFP–2×FYVE_Hrs_ (Figure 4) and GFP–SGK3 (Figure 6) is rapid and essentially complete within 60–120 s, the earliest time point we can reliably assess in live cell imaging experiments. In future work it would be interesting to explore whether there is an interplay between class II and class III PI3Ks and whether inhibition of Vps34 results in the activation of a class II PI3K-driven compensatory mechanism to produce PtdIns(3)*P*. Moreover, we observed a marked reduction in SGK3 activity and phosphorylation within 15 s treatment with VPS34-IN1 which was nearly maximal by 30 s. These observations are consistent with previous work showing that the localization of GFP–2×FYVE_Hrs_ at the endosome is highly dynamic and responsive to inhibition of Vps34 [55,56]. It has been argued that the relatively low affinity of PX and FYVE domains for PtdIns(3)*P* coupled with high myotubularin PtdIns 3-phosphatase activity accounts for rapid and highly dynamic localization and response [25,29]. Our results highlight that full-length SGK3 is similarly dynamic and responsive to changes PtdIns(3)*P* as the well-characterized GFP–2×FYVE_Hrs_ probe. The finding that VPS34-IN1 does not inhibit the localization of the PtdIns(3,4,5)*P*_3_/PtdIns(3,4)*P*_2_ binding GFP–PH_Akt1_ to the plasma membrane or inhibit Akt phosphorylation at Thr^308^ or Ser^473^ establishes that VPS34-IN1 is not suppressing the activity of class I PI3Ks *in vivo*, consistent with the *in vitro* lipid kinase profiling data. The finding that VPS34-IN1 does not suppress activity of SGK2 that does not possess a PtdIns(3)*P*-binding PX domain (Figure 9C) is also consistent with the notion that the ability of VPS34-IN1 to suppress SGK3 is dependent on its ability to bind PtdIns(3)*P*.

SGK3 was first identified as a novel isoform of SGK1 [39] that played a role in enabling the IL (interleukin)-3-dependent survival of haemopoietic cells [57]. Subsequent studies revealed that SGK3 possesses a PtdIns(3)*P*-binding N-terminal PX domain [30,35,36]. To our knowledge SGK3 is unique among kinases in possessing a PtdIns(3)*P*-binding domain. Consistent with previous work [36], we show that endosomal localization, as well as SGK3 activity and phosphorylation of regulatory T-loop and hydrophobic motif, is markedly suppressed following mutation of critical PtdIns(3)*P*-binding PX domain residues. Moreover, inhibiting SGK3 binding to the endosomes by treating cells with VPS34-IN1 induced a rapid 50–60% decrease in SGK3 activity accompanied by a commensurate decrease in the T-loop and hydrophobic motif phosphorylation. In future work it would be important to explore the mechanism by which PtdIns(3)*P* binding and endosomal localization controls SGK3 activity by regulating phosphorylation of its T-loop by PDK1 and hydrophobic motif by mTOR. It would be interesting to explore whether PtdIns(3)*P* binding induces a conformational change in SGK3 promoting phosphorylation by PDK1 and/or mTOR. An analogous mechanism operates for Akt isoforms, in which interaction of the PH domains of these kinases with PtdIns(3,4,5)*P*_3_/PtdIns(3,4)*P*_2_ at the plasma membrane induces a conformational change that markedly enhances phosphorylation and activation by PDK1 [11,12,58,59]. It is also possible that PtdIns(3)*P* binding serves to anchor SGK3 to the endosome, as well as other membranes where a pool of PDK1 and mTOR reside, and this increases the efficiency by which these enzymes are brought together on a two-dimensional membrane surface.

**Figure 10:**
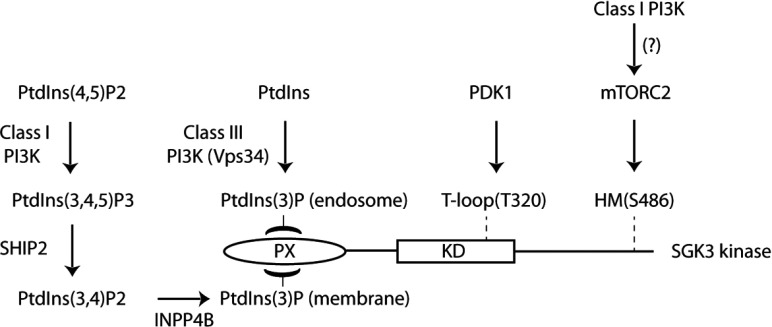
Model of how SGK3 is regulated by two distinct pools of PtdIns(3)*P* Our data suggest that SGK3 is controlled by two pools of PtdIns(3)*P*. One pool is produced via phosphorylation of PtdIns by class III PI3K termed Vps34 at the endosome and the other pool of PtdIns(3)*P* is generated as a result of dephosphorylation of PtdIns(3,4,5)*P*_3_ to PtdIns(3)*P* through the sequential actions of SHIP1/2 and INPP4B PtdIns phosphatases. Our findings suggest that binding of PtdIns(3)*P* to the PX domain triggers SGK3 activation by promoting phosphorylation of the T-loop by PDK1 and the hydrophobic motif by mTOR. Further work is required to understand how binding of PtdIns(3)*P* promotes phosphorylation of SGK3 by PDK1 and mTOR.

Despite mutations inhibiting PtdIns(3)*P* binding suppressing SGK3 activity by over 90%, and VPS34-IN1 treatment dispersing the vast majority of GFP–2×FYVE_Hrs_ and GFP–SGK3 from the endosome, we find that VPS34-IN1 only partially reduced SGK3 activity by 50–60%. Consistent with this, a previous study has found that knockout of Vps34 in mouse embryonic fibroblasts resulted in ~60% loss of PtdIns(3)*P* levels [60], which correlates with the inhibition of SGK3 activity observed in U2OS cells treated with VPS34-IN1. The activity of SGK3 that remains following VPS34-IN1 treatment is likely to be controlled by additional pool(s) of PtdIns(3)*P* generated independently of Vps34 that are able to recruit SGK3 and hence promote activation via mTOR and PDK1. Previous work has revealed that a pool of PtdIns(3)*P* can be generated *in vivo* through the dephosphorylation of PtdIns(3,4,5)*P*_3_ via sequential actions of the PtdIns 5-phosphatases (SHIP1/2) [17] and the PtdIns 4-phosphatase (INPP4B) [18]. PtdIns(3)*P* produced through this route is then rapidly dephosphorylated to PtdIns by a family of myotubularin PtdIns 3-phosphatases [19]. Our finding that treatment of cells with structurally diverse class I selective PI3K inhibitors (GDC-0941 [34] and BKM120 [44]) that do not inhibit Vps34 induce a ~40% reduction in SGK3 activity at doses that suppress Akt activity significantly (Figure 8) suggests that PtdIns(3)*P* generated through dephosphorylation of PtdIns(3,4,5)*P*_3_ could be controlling SGK3. However, we cannot rule out that the effect of GDC-0941 and BKM120 on SGK3 activity in these studies is not at least partially mediated through the ability of these inhibitors to suppress the activity of mTORC2, which is potentially also regulated by class I PI3K [61] (Figure 10).

It is also interesting to note that the impact of GDC-0941 on SGK3 activity is slower than that observed for VPS34-IN1 (compare Figure 9A with 9B), which may be consistent with the several steps between activation of class I PI3K and PtdIns(3)*P*. The observation that combining VPS34-IN1 and GDC-0941 reduces SGK3 activity by 80–90% comparable with the effect of mTOR inhibitors is consistent with the notion that the class I and class III PI3K controlled pools of PtdIns(3)*P* are the key in controlling SGK3 (Figure 10).

In future work it would also be interesting to explore whether residual SGK3 kinase activity (Figure 8A) and the presence of low levels of GFP–2×FYVE_Hrs_ probe and GFP–SGK3 still present on endosomes after dual treatment with class I and class III PI3K inhibitors are due to pools of PtdIns(3)*P* generated by the poorly characterized class II PI3Ks. Previous work has suggested that class II PI3Ks play a role in intracellular vesicular trafficking as the kinases were found to reside on endosomes, clathrin-coated vesicles and *trans*-Golgi network [62,63].

Further work is required to pinpoint the location of the class I PI3K-controlled PtdIns(3)*P* pool in cells. We do not see a marked localization of GFP–2×FYVE_Hrs_ or GFP–SGK3 at the plasma membrane where the class I PI3K controlled the pool of PtdIns(3)*P* might be expected to reside. However, PtdIns(3)*P* at the plasma membrane may be much more dispersed than at the endosome membrane, where PtdIns(3)*P* levels are very high, thereby making GFP–2×FYVE_Hrs_ or GFP–SGK3 localization at the plasma membrane harder to visualize.

There is increasing interest in understanding the physiological roles that Vps34 as well as SGK3 play. Our data indicate that VPS34-IN1 will be a useful tool to probe the cellular functions of Vps34 and that monitoring SGK3 activity and/or phosphorylation could serve as a biomarker for Vps34 activity *in vivo*. There is also increasing evidence that a significant number of human tumours that are insensitive to class I PI3K inhibition display elevated levels of SGK3 and that this plays an important role in driving survival and proliferation [64–68]. In future work it will be important to learn more about the roles that SGK3 plays at the endosome and potentially other membranes. It will be important to identify physiological substrates for SGK3 and explore whether inhibiting Vps34 or SGK3 could be deployed as a therapeutic strategy to treat tumour cells displaying elevated SGK3 activity. Finally, our data indicate that combining GDC-0941 and VPS34-IN1 might be used as a strategy to inhibit pools of PtdIns(3)*P* generated by class I and III PI3Ks that would better allow the study of the roles and regulation of the elusive class II PI3Ks to be visualized.

## Online data

Supplementary data
