# Supplementary material for: Characterization of VPS34-IN1, a selective inhibitor of Vps34, reveals that the phosphatidylinositol 3-phosphate-binding SGK3 protein kinase is a downstream target of class III phosphoinositide 3-kinase
Source: Biochem J. 2014 Oct 10;463(Pt 3):413–27. doi: 10.1042/BJ20140889 (PMC4209782; doi:10.1042/BJ20140889)
Supplement: Supplementary data [file bj4630413ntsadd.pdf]

Supplementary Figure Legends

Figure S1 **IC50 analysis of ability of VPS34-IN1 to inhibit Vps34 kinase employing Life Technology assay**. (A) Recombinant human Vps34 was assayed by measuring phosphorylation of PtdIns in a ^{32}P-radioactive kinase assay by Selectscreen biochemical kinase profiling service at Life technology. The assay conditions are described at www.lifetechnologies.com/selectscreen. . IC_{50} graph was generated using prism software with non-linear regression analysis. Assays were undertaken in duplicates and data presented as mean ± SD and activity plotted relative to 100% activity in DMSO control.

Figure S2 Protein kinase profiling of the VPS34-IN1 at a single concentration of 1μM carried out against the ProQinase panel of 300 protein kinases. Results for each kinase are presented as the mean kinase activity ± SD for an assay undertaken in triplicate relative to a Control kinase assay in which inhibitor was omitted. Abbreviation used for each kinase and assay conditions utilised are defined at the (http://www.proqinase.com)

Figure S3 **IC50 analysis of ability of VPS34-IN1 to inhibit lipid kinases employing ProQinase screen technology.** IC_{50} graphs for PIK3C3, PIP5K1A and PIP5K1C were generated using Prism software with non-linear regression analysis. Abbreviation used for each kinase and assay conditions utilised are defined at the (http://www.proqinase.com

Table S1

Results are presented as the percentage of kinase activity in DMSO control reactions. Protein kinases were assayed *in vitro* in the presence of 1μM VPS34-IN1 inhibitor as described on the International Centre for Kinase Profiling website (http://www.kinase-screen.mrc.ac.uk/) , and the results are means ± S.D. for triplicate reactions. Abbreviations are as follows: ABL, Abelson tyrosine-protein kinase 1; AMPK, AMP-activated protein kinase; ASK, apoptosis signal-regulating kinase; BRK, breast tumour kinase; BRSK, brain-specific kinase; BTK, Bruton''s tyrosine kinase; CaMK, calmodulin-dependent kinase; CaMKK, CaMK kinase; CDK, cyclin-dependent kinase; CHK, checkpoint kinase; CK, casein kinase; CLK, CDC-like kinase; CSK, C-terminal Src kinase; DAPK, death-associated protein kinase; DDR, discoidin domain receptor; DYRK, dual-specificity tyrosine-phosphorylated and regulated kinase; EF2K, elongation-factor-2 kinase; EIF2AK, eukaryotic translation initiation factor 2-alpha kinase; EPH, ephrin; ERK, extracellular signal-regulated kinase; FGF-R, fibroblast growth factor receptor; GCK, germinal centre kinase; GSK, glycogen synthase kinase; HER, human epidermal growth factor receptor; HIPK, homeodomain-interacting protein kinase; IGF1R, IGF1 receptor; IKK, inhibitory κB kinase; IR, insulin receptor; IRAK, interleukin-1 receptor-associated kinase; IRR, insulin-related receptor; JAK, Janus kinase; JNK, c-Jun N-terminal kinase; Lck, lymphocyte cell-specific protein tyrosine kinase; LKB1, liver kinase B1; MAPK, mitogen-activated protein kinase; MAPKAP-K, MAPK-activated protein kinase; MARK, microtubule-affinityregulating kinase; MEKK, MAP kinase kinase kinase; MELK, maternal

embryonic leucinezipper kinase; MINK, misshapen/NIK-related kinase; MKK, MAPK kinase; MLK, mixed lineage kinase; MNK, MAPK-integrating protein kinase; MPSK, myristoylated and palmitoylated serine/threonine-protein kinase; MSK, mitogen- and stress-activated protein kinase; MST, mammalian homologue Ste20-like kinase; NEK, NIMA (never in mitosis in *Aspergillus nidulans*)-related kinase; NUAK, novel (NUA) family SnF1-like kinase; OSR, oxidative stress-responsive kinase; PAK, p21-activated protein kinase; PDGFRA, platelet-derived growth factor receptor-α; PDK, phosphoinositide-dependent kinase; PHK, phosphorylase kinase; PIM, provirus integration site for Moloney murine leukaemia virus; PINK (insect homologue), PTEN-induced kinase; PKA, cAMP-dependent protein kinase; PKB, protein kinase B; PKC, protein kinase C; PKD, protein kinase D; PLK, polo-like kinase; PRAK, p38-regulated activated kinase; PRK, protein kinase C-related kinase; RIPK, receptor-interacting protein kinase; ROCK, Rho-dependent protein kinase; RSK, ribosomal S6 kinase; S6K, p70 ribosomal S6 kinase; SGK, serum- and glucocorticoid-induced protein kinase; SIK, salt-induced kinase; smMLCK, smooth muscle myosin light-chain kinase; SRPK, serine/arginine protein kinase; STK, serine/threonine kinase; SYK, spleen tyrosine kinase; TAK, TGFβ-activated kinase; TAO, thousand and one amino acid; TBK1, TANK-binding kinase 1; TESK, testis-specific protein kinase; TGFBR, TGFβ receptor; TIE, tyrosine-protein kinase receptor; TLK, tousled-like kinase; TrkA, tropomyocin receptor kinase; TSSK, testis-specific serine/threonine-protein kinase; TTBK, tau-tubulin kinase; ULK, Unc-51-like kinase; VEGFR, vascular endothelial growth factor receptor; WNK, with no lysine; YES1, Yamaguchi sarcoma viral oncogene homologue 1; ZAP, ζ-chain-associated protein.

FIGURE S1

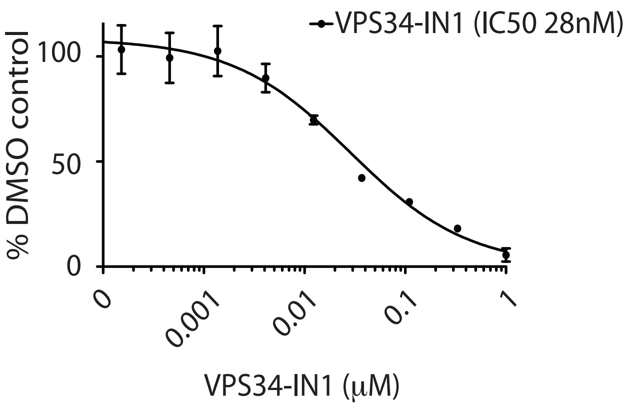

VPS34-IN1 (IC50 28nM)
% DMSO control
100
50
0
0
0.001
0.01
0.1
1
VPS34-IN1 (μM)

FIGURE S2

VPS34-IN1, 1μM (ProQinase panel)

VPS34-IN1, 1μM (ProQinase panel)

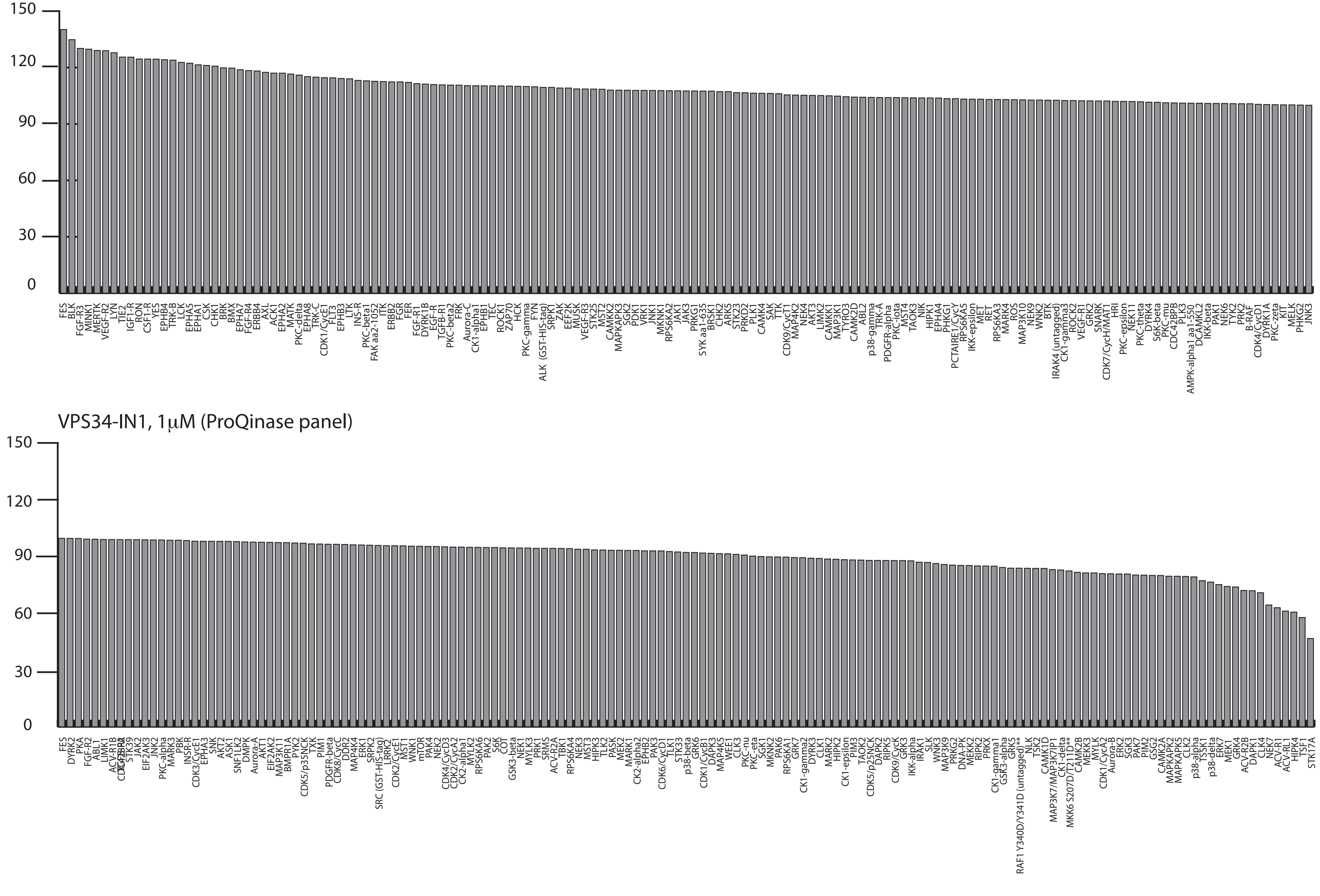

FIGURE S3

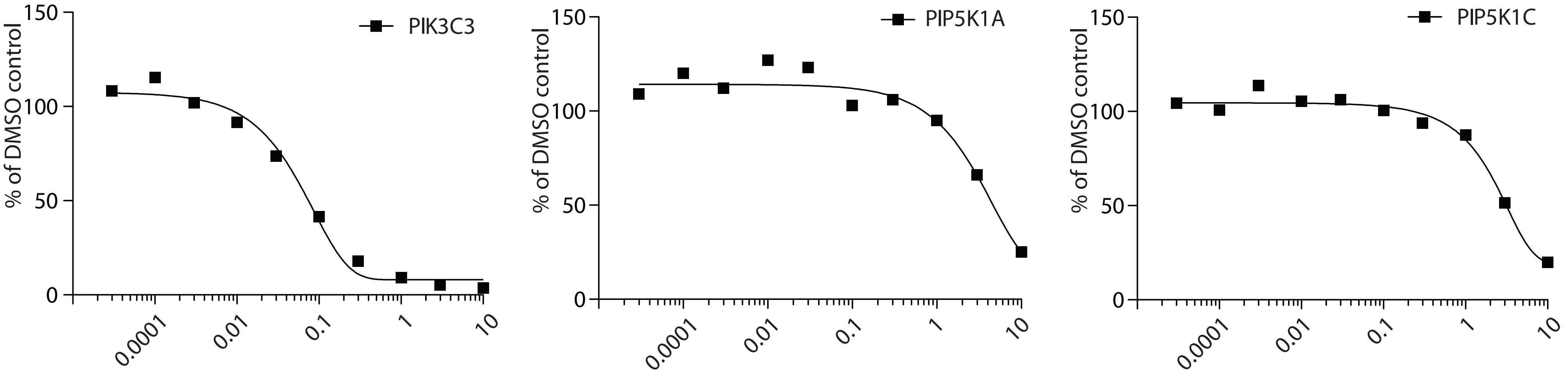

% of DMSO control
150
100
50
0
PIK3C3
0.0001
0.01
0.1
1
10
% of DMSO control
150
100
50
0
PIP5K1A
0.0001
0.01
0.1
1
10
% of DMSO control
150
100
50
0
PIP5K1C
0.0001
0.01
0.1
1
10

Table S1

p38g MAPK	133
IRAK4	133
CSK	132
MLK3	129
TBK1	125
p38a MAPK	125
EIF2AK3	125
ASK1	124
MKK6	124
STK33	124
PKBb	123
TGFBR1	123
MNK1	122
CK1γ2	122
MKK1	122
PDGFRA	121
MST2	120
MLK1	119
PKCa	119
MST3	118
ULK2	118
MPSK1	118
TSSK1	118
CHK1	118
HIPK3	117
MAP4K3	117
PRK2	116
CAMK1	116
WNK1	116
S6K1	116
PKBa	116
ERK2	116
MAPKAP-K3	115
EF2K	115
p38b MAPK	115
MARK4	115
PKD1	115
MARK1	114
PLK1	114
OSR1	114
PIM3	114
JNK2	113
PIM2	113
VEG-FR	113
IRR	113
IRAK1	112
PKA	112
NUAK1	112
DYRK3	112
SIK3	112
JNK1	112
p38d MAPK	111
MKK2	111
EPH-B3	111
ROCK 2	111
CK2	111
SRPK1	111
DDR2	111
PAK2	111
PKCγ	111
MAPKAP-K2	110
Aurora A	110
ZAP70	110
IR	109
MEKK1	109
PINK	109
MARK3	109
DYRK2	109
EPH-B2	109
BRSK1	109
PIM1	108
TTK	108
CAMKKb	108
BTK	108
MELK	108
MARK2	108
HIPK1	108
SGK1	108
ERK1	108
MST4	108
LKB1	106
EPH-B4	106
IKKb	106
SIK2	106
TTBK1	106
MAP4K5	105
PDK1	105
DYRK1A	105
CK1δ	105
MSK1	105
PHK	105
PRAK	105
ERK5	104
GCK	104
PAK4	103
RSK2	103
PKCz	103
CHK2	103
NEK2a	103
MINK1	103
IKKe	103
BRK	103
IGF-1R	101
EPH-A2	101
HER4	101
MNK2	100
TLK1	100
RSK1	99
EPH-B1	99
NEK6	98
ULK1	98
BRSK2	98
SmMLCK	97
PAK6	97
AMPK (hum)	96
PAK5	96
DAPK1	96
HIPK2	96
JNK3	95
FGF-R1	95
TESK1	95
TIE2	93
EPH-A4	93
JAK2	93
Lck	92
SYK	91
CDK9-Cyclin T1	91
TTBK2	88
CDK2-Cyclin A	88
ABL	87
Src	84
Aurora B	83
CLK2	82
TAO1	80
RIPK2	79
YES1	77
TrkA	70
GSK3b	51
TAK1	40
ERK8	36
